# Repurposing disulfiram, an alcohol-abuse drug, in neuroblastoma causes KAT2A downregulation and in vivo activity with a water/oil emulsion

**DOI:** 10.1038/s41598-023-43219-2

**Published:** 2023-09-30

**Authors:** Annie Beaudry, Simon Jacques-Ricard, Anaïs Darracq, Nicolas Sgarioto, Araceli Garcia, Teresita Rode García, William Lemieux, Kathie Béland, Elie Haddad, Paulo Cordeiro, Michel Duval, Serge McGraw, Chantal Richer, Maxime Caron, François Marois, Pascal St-Onge, Daniel Sinnett, Xavier Banquy, Noël J.-M. Raynal

**Affiliations:** 1 Sainte-Justine University Hospital Research Center, 3175, Chemin de la Côte-Sainte-Catherine, Montreal, QC H3T 1C5 Canada; 2 Département de Pharmacologie et de Physiologie, Faculté de Médecine, Université de Montréal, Montreal, QC Canada; 3 Faculté de Pharmacie, Université de Montréal, Montreal, QC Canada; 4 Département de Pédiatrie, Université de Montréal, Montreal, QC Canada

**Keywords:** Cancer, Drug discovery

## Abstract

Neuroblastoma, the most common type of pediatric extracranial solid tumor, causes 10% of childhood cancer deaths. Despite intensive multimodal treatment, the outcomes of high-risk neuroblastoma remain poor. We urgently need to develop new therapies with safe long-term toxicity profiles for rapid testing in clinical trials. Drug repurposing is a promising approach to meet these needs. Here, we investigated disulfiram, a safe and successful chronic alcoholism treatment with known anticancer and epigenetic effects. Disulfiram efficiently induced cell cycle arrest and decreased the viability of six human neuroblastoma cell lines at half-maximal inhibitory concentrations up to 20 times lower than its peak clinical plasma level in patients treated for chronic alcoholism. Disulfiram shifted neuroblastoma transcriptome, decreasing MYCN levels and activating neuronal differentiation. Consistently, disulfiram significantly reduced the protein level of lysine acetyltransferase 2A (KAT2A), drastically reducing acetylation of its target residues on histone H3. To investigate disulfiram’s anticancer effects in an in vivo model of high-risk neuroblastoma, we developed a disulfiram-loaded emulsion to deliver the highly liposoluble drug. Treatment with the emulsion significantly delayed neuroblastoma progression in mice. These results identify KAT2A as a novel target of disulfiram, which directly impacts neuroblastoma epigenetics and is a promising candidate for repurposing to treat pediatric neuroblastoma.

## Introduction

Neuroblastomas are pediatric tumors that arise from sympathetic neurons in the adrenal glands, the paraspinal area, or the pelvic ganglia and consist of small, round, undifferentiated cells called neuroblasts. Neuroblastoma is the most common extracranial solid tumor in children, and is responsible for 10% of deaths from childhood cancers^1–5^. It has diverse clinical presentations, ranging from complete regression with minimal treatment to aggressive metastatic disease with poor prognosis^6^. Approximately half of those with neuroblastoma develop high-risk tumors that metastasize to the lymph nodes, bone marrow, bone, liver, skin, and more rarely, the lungs and brain^5^. In the last few decades, the implementation of intensive multimodal therapy has increased the 5-year survival rate of children with metastatic neuroblastoma from < 20 to > 50%^7^. High-risk neuroblastoma is now treated via surgical resection, myeloablative chemotherapy with hematopoietic stem cell transplantation, then adjuvant retinoid differentiation treatment and immunotherapy with anti-disialoganglioside-2 monoclonal antibodies. However, these children still have poor outcomes and new treatments that can be rapidly tested in clinical trials and are safe for child development are urgently needed.

Aggressive neuroblastoma is associated with frequent genetic alterations, such as loss of chromosome 1p and 11q, gain of 17q, and amplification of *MYCN* proto-oncogene, a basic helix-loop-helix transcription factor^6^. The latter is found in 20% of primary neuroblastomas and 50% of high-risk tumors^2–6,8,9^. MYCN is a transcription factor that regulates a myriad of cellular processes, such as proliferation, metabolism, and differentiation^10^. In contrast to other MYC family transcription factors, MYCN is expressed exclusively during embryogenesis and only in specific cell types (such as pre-B cells, kidney, forebrain, hindbrain, and intestinal cells)^10^. MYCN overexpression transforms normal neuroectodermal cells into neuroblasts by stimulating the expression of growth-promoting genes^10–14^. MYCN impacts the transcription of target genes by recruiting histone lysine acetyltransferases (KATs), which acetylate promoter regions to open the chromatin structure and favor gene expression^10,15,16^. MYC family of transcription factors are associated with several epigenetic regulators during development and cancer, including KAT2A (also known as GCN5). KAT2A is part of the multiprotein transcriptional coactivator SAGA (*S*pt-*A*da-*G*cn5-*A*cetyltransferase) and acetylates lysine residues on histones in gene promoter regions^17–19^. Interestingly, KAT2A can also acetylate non-histone proteins, including MYCN. Acetylation increases MYCN’s half-life, enhancing its effects on its target genes^19^.

Pharmacological interventions directly targeting MYCN have been unsuccessful so far. Alternative approaches to downregulate MYCN’s activity in cancer target proteins involved in its oncogenic signalling pathways, such as bromodomain family members^20^. Targeting KAT2A could provide a new strategy to block MYCN’s oncogenic activity in neuroblastoma. Several structurally different KAT inhibitors exist, including natural products, synthetic small molecules and peptides. However, although they induce hypoacetylation in preclinical studies^19,21,22^, their clinical efficacies have yet to be tested.

Drug development for children raises major concerns regarding long-term safety and toxicity, particularly for children with cancer, who typically endure several cycles of intensive therapies. Thus, new drug approvals in pediatric oncology usually occur only after drugs have been developed and approved for adults^23^. Given the safety concerns and the cost of developing drugs for rare diseases, there is high interest in repurposing drugs approved for other clinical indications to treat childhood cancers. Compared to developing an entirely new drug, drug repurposing has several advantages: it is cheaper, accelerates the path to clinical trials, and safe, which is particularly important for children^24^. While screening US Food and Drug Administration (FDA)-approved drugs, we discovered a series of drugs with previously unknown epigenetic activities in cancer cells^25,26^. When we examined their potencies against neuroblastoma cell lines with and without *MYCN* amplification, one approved drug stood out: disulfiram (tetraethylthiuram disulfide; sold under the trade name Antabuse). Approved to treat chronic alcoholism in 1951^27,28^, disulfiram irreversibly inhibits aldehyde dehydrogenase (ALDH), blocking the conversion of acetaldehyde to acetic acid—which has no unpleasant effects unless alcohol is consumed. Disulfiram’s repurposing potential for oncology is being actively pursued. Its tumor suppressive effects are likely mediated via several targets, including ALDH (which is overexpressed in cancer stem cells)^29,30^, as well as NPL4 homolog ubiquitin recognition factor, the proteasome, and oxidative stress signaling^31–33^. Disulfiram’s metabolism and toxicological profile are well known. It is > 80% bioavailable after oral administration, and 20% remains in the body for 1–2 weeks. The typical dose to treat adults for chronic alcoholism is 500 mg/day, which produces a 20 µM peak plasma concentration^27,28^. Importantly, long-term disulfiram use is safe, and could actually be cancer preventive—in one study, patients with cancer under continuous disulfiram treatment had a lower risk of death from cancer than those who stopped taking disulfiram at cancer diagnosis^32^. When disulfiram and its active metabolite, sodium diethyldithiocarbamate trihydrate (DDTC) were tested in clinical trials as adjuvant treatments in adults with non-small cell lung cancer and high-risk breast cancer, respectively, both trials demonstrated improvement in overall survival^34,35^. Thus, disulfiram is a promising candidate to repurpose for oncology.

In this study, we have assessed disulfiram’s effects on neuroblastoma using in vitro and in vivo models, focusing on how it modulates the neuroblast transcriptome and epigenome, particularly via MYCN and KAT2A. To enable disulfiram’s intraperitoneal injection into mice and overcome its low solubility and instability in water, we developed an optimized water-in-oil (W/O) emulsion that delivers disulfiram with adequate viscosity. This formulation is presented as a proof of concept to establish the feasibility of repurposing disulfiram to treat pediatric neuroblastoma tumors.

## Results

### Disulfiram has anticancer effects against neuroblastoma cell lines in the nanomolar range

**Figure 1:**
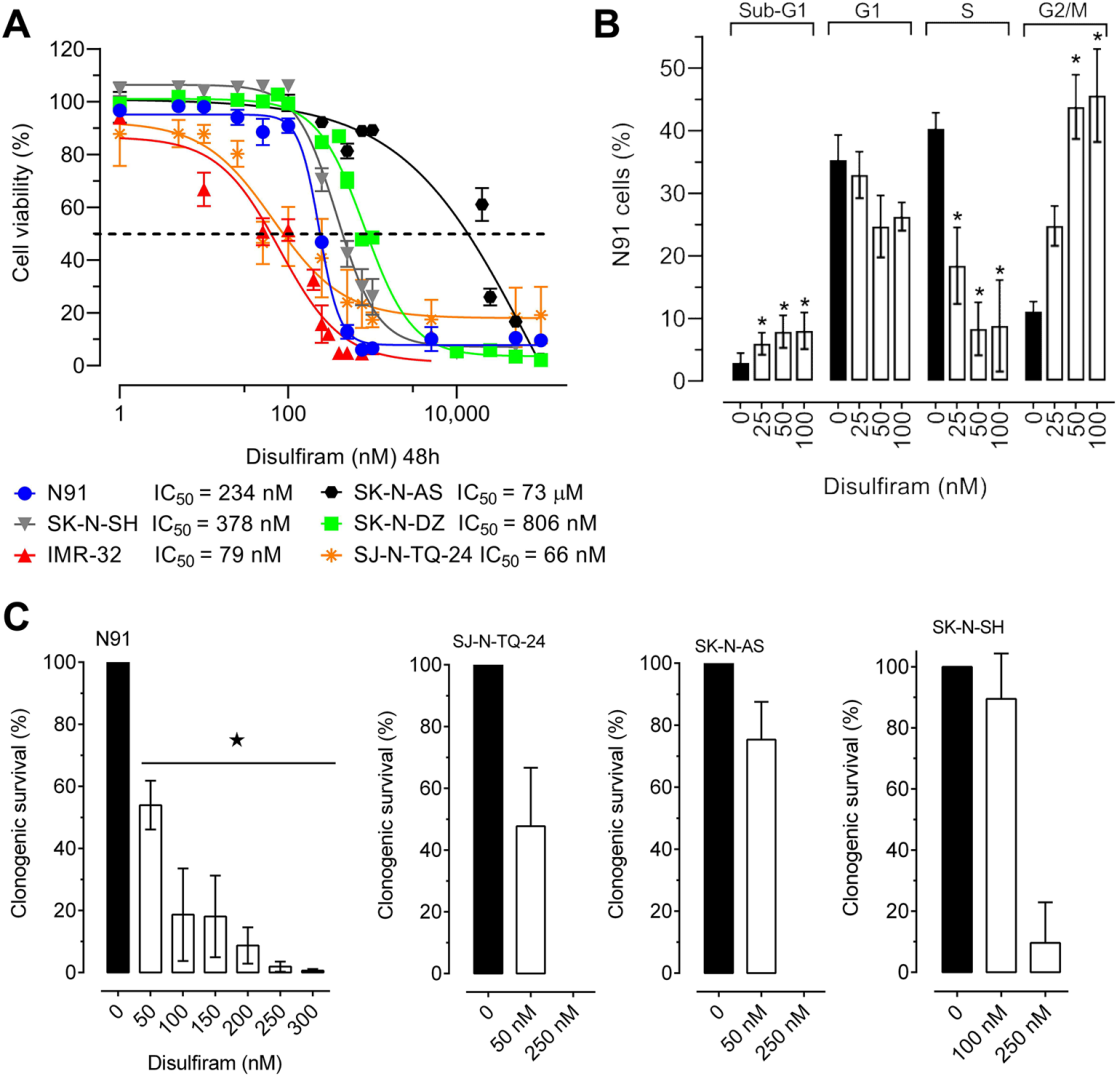
Disulfiram has anticancer effects on neuroblastoma cell lines. (**A**) The viabilities of *MYCN-*amplified cells (SK-N-DZ, N91, SJ-N-TQ-24 and IMR-32) and non-amplified cells (SK-N-AS and SK-N-SH) were measured after 48 h of treatment with disulfiram (1 nM to 100 µM) or DMSO (*n* = 3). IC_50_ values are shown below the graph. (**B**) Cell cycle analysis of N91 cells treated with 25, 50 and 100 nM disulfiram for 48 h (*n* = 3). (**C**) Clonogenic assays in N91, SJ-N-TQ-24, SK-N-AS and SK-N-SH cells after 48 h disulfiram exposure at the indicated concentrations. After treatment, the drug was removed, and fresh cell culture medium (without drug) was added. Colony formation was measured after 2 weeks. Stars indicate significant differences between the treated group and the DMSO control (*p* ≤ 0.05 by unpaired Student’s *t*-test).

We tested the anticancer effects of disulfiram in four neuroblastoma cell lines with *MYCN* amplification (IMR-32, SK-N-DZ, SJ-N-TQ-24 and IGR-N91, hereafter named N91) and two without (SK-N-AS and SK-N-SH; note that these lines do express high levels of the oncogene *MYC,* according to depmap.org). Neuroblast viability was evaluated in response to 48 h disulfiram exposure (Fig. 1A). The most sensitive *MYCN*-amplified cell lines were IMR-32 and SJ-N-TQ-24 with IC_50_ values of 79 and 66 nM, respectively. The other *MYCN*-amplified lines, SK-N-DZ and N91, had IC_50_ values of 806 nM and 234 nM, respectively, while the IC_50_ values of the non-*MYCN* amplified neuroblastoma cells SK-N-AS and SK-N-SH were 73 µM and 378 nM, respectively. Interestingly, 5/6 neuroblastoma lines exhibited sensitivity to disulfiram at levels 25–300 times lower than the peak plasma concentration (20 µM) measured in patients with chronic alcoholism disulfiram treatment^36^.

To better understand the short-term effects of disulfiram on neuroblasts, we performed cell cycle analysis on the *MYCN*-amplified N91 line after 48 h of exposure to nanomolar concentrations (Fig. 1B). The sub-G1 cell population was significantly increased by all doses; however, it comprised < 10% of the cells even at the highest dose. Disulfiram did not significantly change the proportion of cells in G1 but significantly decreased the proportion of S phase cells at all doses. This was accompanied by increases in G2/M cells, which were significant at 50 and 100 nM. Cell cycle changes were also observed in SK-N-SH cells (without *MYCN* amplification) after disulfiram treatment (Suppl. Fig. S1A). Thus, disulfiram produced anticancer effects after short-term exposure. Cell cycle block mediated by disulfiram occurred at lower doses than the reduction in cell viability, suggesting that more time may be needed to observe disulfiram’s anticancer effects. As disulfiram has been associated with reduced cancer stem cell renewal in adult solid tumors^29,30^, we evaluated its effects in long-term clonogenic assays on neuroblast renewal (Fig. 1C). *MYCN-*amplified N91 and SJ-N-TQ-24 neuroblasts lost their clonogenic potential at doses of approximately 300 nM. Similar results were observed in non-*MYCN*-amplified SK-N-AS and SK-N-SH cells. Low doses used cell cycle analysis depict similar activities as observed in long-term clonogenic assays. Altogether, these results showed that disulfiram reduced neuroblastoma proliferation and abolished clonogenicity at concentrations well below clinical plasma levels, suggesting repurposing potential for neuroblastoma.

### Disulfiram shifts the neuroblast transcriptome

**Figure 2:**
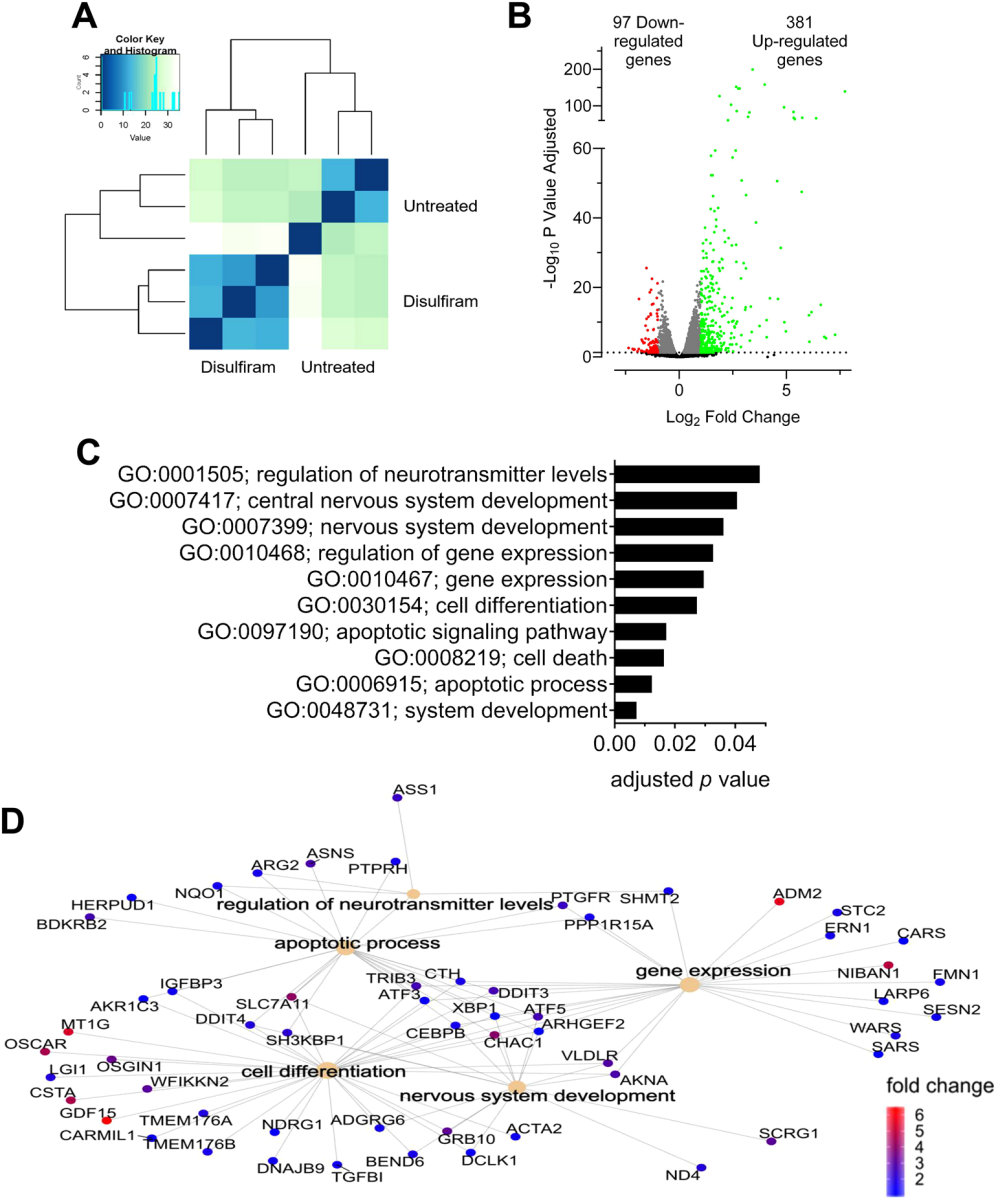
Disulfiram modifies the transcriptomes of neuroblastoma cells. *MYCN*-amplified N91 cells were treated with disulfiram (50 nM for 48 h) and analyzed by RNA sequencing. (**A**) Heat map of similarities between corresponding reads per kilobase million (RPKM) values in untreated and disulfiram-treated N91 cells (*n* = 3). Green indicates high similarity and blue indicates low similarity. (**B**) Transcriptome changes induced by disulfiram are shown in a volcano plot to illustrate the distributions of significantly regulated genes (adjusted* p* value ≤ 0.05) and unaffected genes (black dots). Grey dots represent genes with adjusted *p* values ≤ 0.05 but with fold change differences between −1 and 1. Downregulated genes with adjusted* p* values ≤ 0.05 and fold changes < −1 are indicated by red dots. Upregulated genes with adjusted* p* values ≤ 0.05 and fold changes > 1 are shown in green. The numbers of downregulated and upregulated genes are shown on the graphs. (**C**) Metascape analysis of genes significantly upregulated by disulfiram. The top 10 GO terms are listed. (**D**) Fold changes in the expression in a subset of genes annotated to the GO terms listed.

We previously reported that disulfiram induced gene reactivation in colon cancer cells^25,26^. Thus, we investigated disulfiram’s transcriptomic effects on *MYCN*-amplified N91 cells by RNA sequencing (Fig. 2A; *n* = 3; data are available in the Gene Expression Omnibus database under accession number GSE226162). After low dose disulfiram treatment (50 nM for 48 h), 97 genes were significantly downregulated (Fig. 2B**,** log_2_ fold change < -1; adjusted* p* value < 0.05) and 381 were significantly upregulated (Fig. 2B**,** log_2_ fold change > 1; adjusted* p* value < 0.05). The upregulated genes were enriched for gene ontology (GO) terms related to nervous system development and cell differentiation, suggesting that these changes influence neuroblast identity (Fig. 2C). Interestingly, we observed neurite outgrowth in the N91 colonies 2 weeks after disulfiram treatment (by three-fold), suggesting differentiation-related morphological changes (Suppl. Fig. S1B). G2/M arrest (Fig. 1B) was confirmed by downregulation of cell cycle essential genes (Suppl. Fig. S1C). Pathways regulating cell death and particularly apoptosis were also upregulated (Fig. 2C, D), confirming the loss of viability observed after treatment (Fig. 1A, B). The downregulated GO terms were enriched in ion homeostatis and substrate specific transporter activity, as we reported in colon cancer cells (Suppl. Fig. S1D)^37^. The interconnectivity of upregulated genes linked to the enriched GO terms suggests that disulfiram induced the expression of genes that influence both cell differentiation and nervous system development (Fig. 2D). Altogether, transcriptomic analysis revealed that disulfiram treatment activated both nervous system differentiation and cell death pathways, consistent with the phenotypic changes observed in the neuroblasts.

### Disulfiram downregulates neuroblast KAT2A, histone H3 acetylation, and MYCN levels

**Figure 3:**
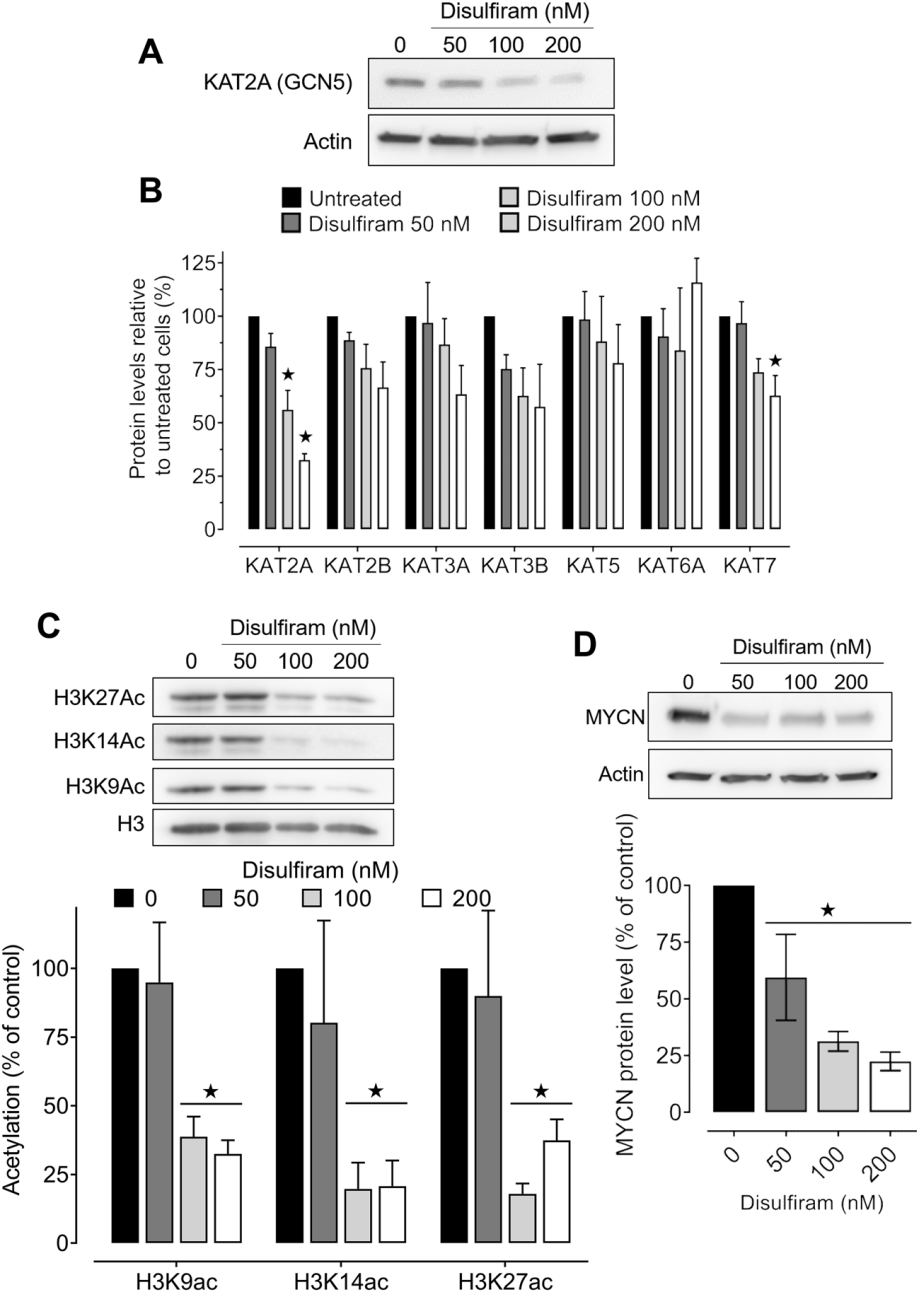
Disulfiram downregulates KAT2A, histone H3 acetylation, and MYCN in neuroblastoma cells. *MYCN*-amplified N91 neuroblasts were exposed to 50, 100, or 200 nM disulfiram treatment for 48 h. (**A**) Representative western blots of KAT2A in untreated and disulfiram-treated cells. Actin was used as a loading control. (**B**) The relative protein levels of seven histone acetyltransferases in untreated and disulfiram-treated cells, normalized to actin levels. (**C**) Acetylation levels on lysine residues 9, 14 and 27 of histone H3 relative to the total H3 level. (**D**) MYCN expression levels in untreated and disulfiram-treated cells relative to actin levels. Stars indicate significant differences from the untreated control (*p* ≤ 0.05, by unpaired Student’s *t*-test).

As we previously identified disulfiram’s epigenetic effects on cancer cells^25,26^, we hypothesized that epigenetic modifications were causing the observed gene expression changes in disulfiram-treated neuroblasts. We previously demonstrated that disulfiram treatment did not affect DNA methylation^25^; therefore, we explored its effects on the histones. Since the transcriptomic responses occurred rapidly after disulfiram treatment, we investigated changes in histone acetylation, which quickly adapts to cellular signals and drug treatments^38^. We first measured the protein levels of seven KATs (Fig. 3A, B) and two histone deacetylases (HDACs; Suppl. Fig. S2A) by western blotting. KAT2A was significantly and dose-dependently decreased after exposure to 100 and 200 nM disulfiram, while KAT7 was decreased only at the highest dose (Fig. 3B). HDAC1 and HDAC2 protein levels were not significantly altered by disulfiram treatment (Suppl. Fig. S2A).

Given the decrease in KAT2A, we next asked whether its target lysine residues on histone H3 (H3K9, H3K14, and H3K27^39^) were impacted by disulfiram treatment. All three acetylation marks were significantly downregulated after a 48 h exposure to disulfiram (100–200 nM; Fig. 3C). Conversely, histone methylation on lysines 9 and 27 (H3K9me2, H3K9me3, H3K27me2, H3K27me3) was unchanged after disulfiram treatment (Suppl. Fig. S2B). Since KAT2A contributes to MYCN oncogenic signaling where its acetylation increases its protein stability, we measured MYCN levels by western blotting after disulfiram exposure (48 h, Fig. 3D). Interestingly, disulfiram significantly decreased the protein level of MYCN, which is the main oncogenic driver of high-risk neuroblastoma. MYCN mRNA levels were also downregulated after treatment in our RNA sequencing dataset (Suppl. Fig. S2C). In addition, several MYCN target genes (curated in the ChIP Enrichment Analysis database^40^) were downregulated after disulfiram treatment, with six genes significantly downregulated (log_2_ fold change < −1, adjusted* p* value < 0.05) and 52 more moderately downregulated (log_2_ fold changes between −0.5 and −1, adjusted* p* value < 0.05; Suppl. Table S1)^40,41^. The reduction in MYCN levels induced by low dose disulfiram treatment correlated with the increase in neuronal differentiation pathways revealed by RNA-seq (Fig. 2). The onset of neuronal differentiation may be responsible for the loss of clonogenic potential after low dose treatment in long-term experiments (Fig. 1C). Altogether, these results demonstrate that disulfiram downregulates KA2TA, decreasing histone acetylation and MYCN levels in neuroblastoma cells.

### Formulation of emulsified disulfiram for in vivo studies

Targeting MYCN overexpression to treat neuroblastoma has been challenging, and repurposing disulfiram in this context may be a game changer. Thus, we next evaluated disulfiram in a preclinical mouse model of neuroblastoma. Disulfiram’s lipophilicity requires a different formulation for mice than for in vitro studies, in which it was dissolved in DMSO. We therefore sought to develop an emulsion loaded with sufficient disulfiram to treat neuroblastoma in preclinical studies. To achieve this, we first characterized the physical and chemical stabilities of various unloaded emulsions of water and soybean oil to investigate their emulsification capacity without the influence of disulfiram. We next characterized the drug’s incorporation into the oil phase of satisfactory formulations. We used dye solubility assays to confirm the formation of W/O emulsions (data not shown).

We tested surfactant mixtures with hydrophilic-lipophilic balance (HLB) values of 5–13 (where 5 indicates a hydrophobic mixture and 13 a hydrophilic mixture), which were calculated according to an established formula^42^. We observed that stable emulsions had HLB values of 5.4 and 6.4. Then, we next monitored disulfiram’s stability in emulsions with different oil–water ratios (50:50, 55:45, 60:40, and 62:38) at room temperature (RT; 20 °C) and 4 °C by high-performance liquid chromatography coupled to an ultraviolet detector (HPLC–UV; Suppl. Fig. S3A, B). The 62:38 emulsion maintained the highest disulfiram stability at RT and 4 °C for at least 1 week.

To monitor the creaming, sedimentation, flocculation, and coalescence of the loaded emulsions, we followed phase separation visually (Suppl. Fig. S4A–C)^43^. Consistent with the disulfiram stability results, formulation F3 (the 62:38 ratio) was the most stable over the week at RT and at 4 °C, with barely noticeable phase separation before day 2, which increased until day 6. Therefore, we used F3 to generate a disulfiram-loaded emulsion for comparison. Emulsion stability was similar with and without disulfiram at RT and at 4 °C over 3 days (Suppl. Fig. S4B). The obtained particles were mostly 10–15 µm, with 40% of particles with a size of 10 µm for the blank and 10–15 µm for the loaded emulsion (Suppl. Fig. S4C). This particle size range is expected for a stable emulsion undergoing a slow separation process, consistent with our visual phase separation observations.

Emulsion viscosity was monitored to ensure reproducible production and stable storage over time (Suppl. Fig. S5A)^44^. Higher viscosity correlates with higher visual stability (*i.e*., the absence of phase separation) but could be problematic for peritoneal administration. As oil is the continuous phase, it conditions the viscosity of our emulsion. Both the blank and loaded F3 emulsions had viscosities of approximately 450 centipoises (cP), suggesting similar rheological behavior despite the presence of disulfiram. F4 (the 60:40 ratio) had the highest viscosity (705 cP), which may be unsuitable for peritoneal administration. F1 and F2 both had low viscosities (132 cP and 83 cP, respectively). This is consistent with their observed low stability, with F2 slightly less stable than F1.

**Figure 4:**
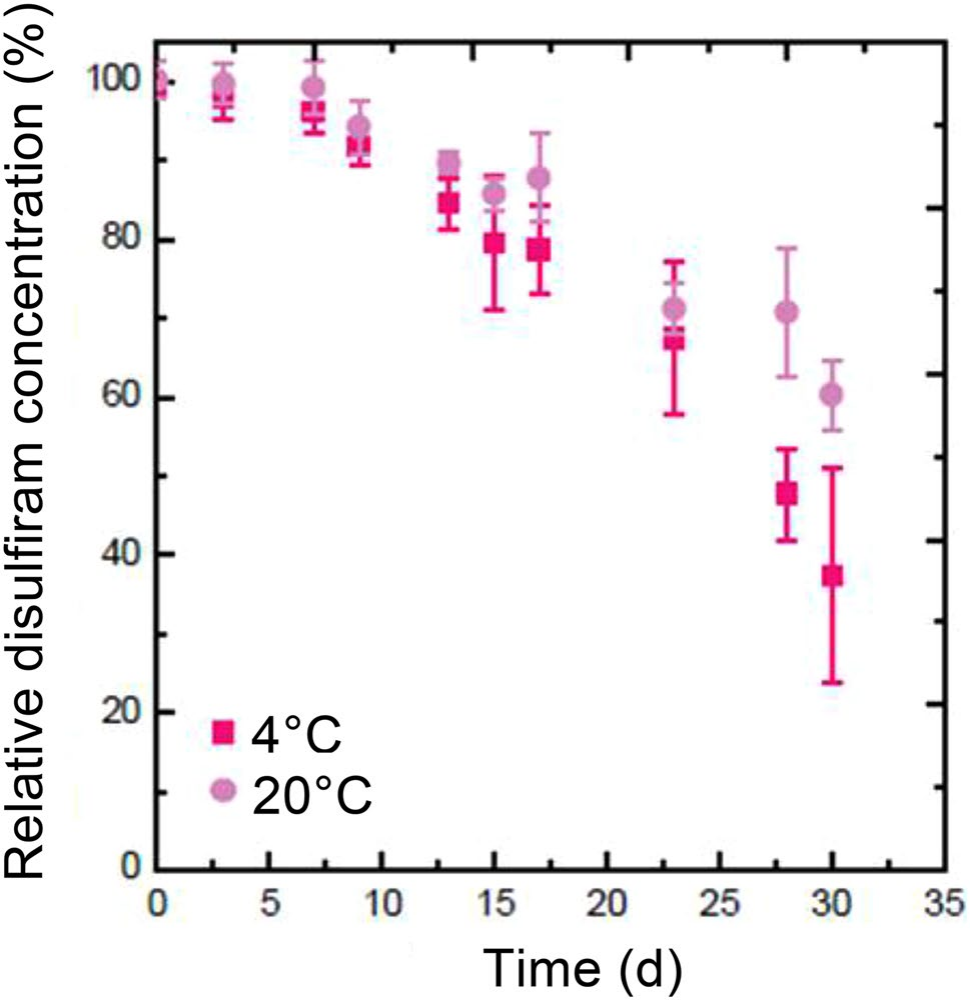
Stability of the disulfiram W/O emulsion. Disulfiram’s chemical stability was measured by HPLC–UV at 4 °C and room temperature (20 °C) for 30 days.

For preclinical use, it will be important for the emulsion to maintain a pH near 7.4. All formulations were neutral immediately after production and stable for > 1 week (data not shown), making them compatible with injection into animals. Finally, the long-term chemical stability of disulfiram in F3 was assessed by HPLC–UV at 4 °C and RT for 30 days. Disulfiram was > 95% stable over the first 3 days (Fig. 4). After 1 week in the emulsion, its stability decreased and it began to precipitate and form crystals, reducing the emulsion’s disulfiram concentration. Importantly, no degradation products were detected in HPLC–UV chromatograms over the course of the stability test. Overall, these results demonstrate that a 62:38 ratio W/O disulfiram emulsion is stable and has excellent physicochemical properties for in vivo administration.

### In vitro disulfiram release from the emulsion

**Figure 5:**
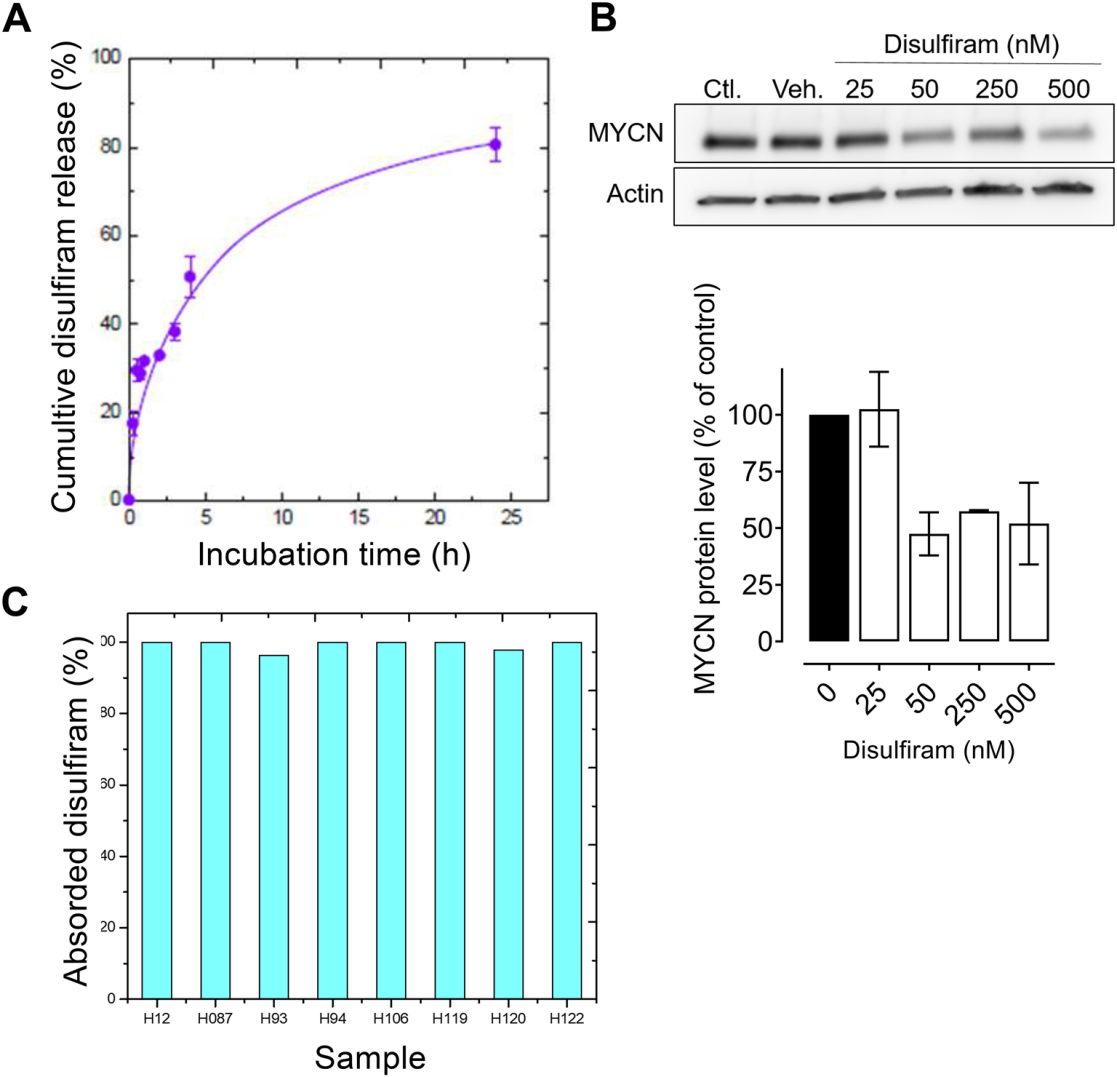
Disulfiram release from the emulsion in vitro and in vivo. (**A**) The disulfiram release profile from the developed 62:38 emulsion at room temperature (mean ± standard error of the mean, *n* = 3). (**B**) *MYCN*-amplified N91 neuroblastoma cells were treated with 62:38 emulsions containing 25, 50, 250 and 500 nM disulfiram for 48 h. Representative western blots for MYCN and actin in untreated N91 cells (Ctl) and those exposed to empty (Veh.) or disulfiram-loaded emulsions (*n* = 2) and quantified MYCN levels relative to actin are shown. (**C**) Disulfiram absorption in the intraperitoneal liquid of treated mice (*n* = 8) was measured by HPLC–UV.

We next assessed the bioavailability of emulsified disulfiram, i.e., its transfer from the W/O emulsion into a bulk aqueous phase (Fig. 5A). Disulfiram release was relatively slow, nearing 80% after 20 h. Before testing the loaded emulsion in mice, we performed a cell culture assay to determine whether disulfiram emulsion administration could induce MYCN downregulation, as it did in DMSO (Fig. 3D). Interestingly, emulsified disulfiram significantly decreased the MYCN level, reducing it by half compared to untreated cells exposed to DMSO or vehicle emulsion (Fig. 5B). This demonstrated that emulsified disulfiram is bioavailable and active in neuroblastoma cells.

### Emulsified disulfiram was almost completely absorbed from the intraperitoneal fluid of treated animals

We used high-resolution mass spectrometry (HR-MS) to quantify the remaining disulfiram and identify metabolites in the oil phase of intraperitoneal fluid samples from treated animals. Residual disulfiram was detected in only 2/8 animals (H067, H120), which represented < 3% of the initial administrated dosage (Fig. 5C). In the remaining six samples, the concentration of disulfiram was not detectable. The known exact masses of disulfiram metabolites^45^ were compared with those detected by HR-MS. In the identification and the elucidation strategies, procedural blank measurements were used to ensure that the substance did not arise from sample preparation or instrument analysis. Disulfiram was rapidly reduced via characteristic disulfide reactions to yield the thiol DDTC, which was metabolized to the corresponding *S*-linked glucuronide and two *S*-methylated compounds (*S*-methyl-*N,N*-diethyldithiocarbamate and *S*-methyl-*N,N*-diethylthiocarbamate; data not shown). Altogether, the data showed that emulsified disulfiram was well absorbed.

### In vivo disulfiram treatment inhibits *MYCN*-amplified neuroblastoma growth in mice

**Figure 6:**
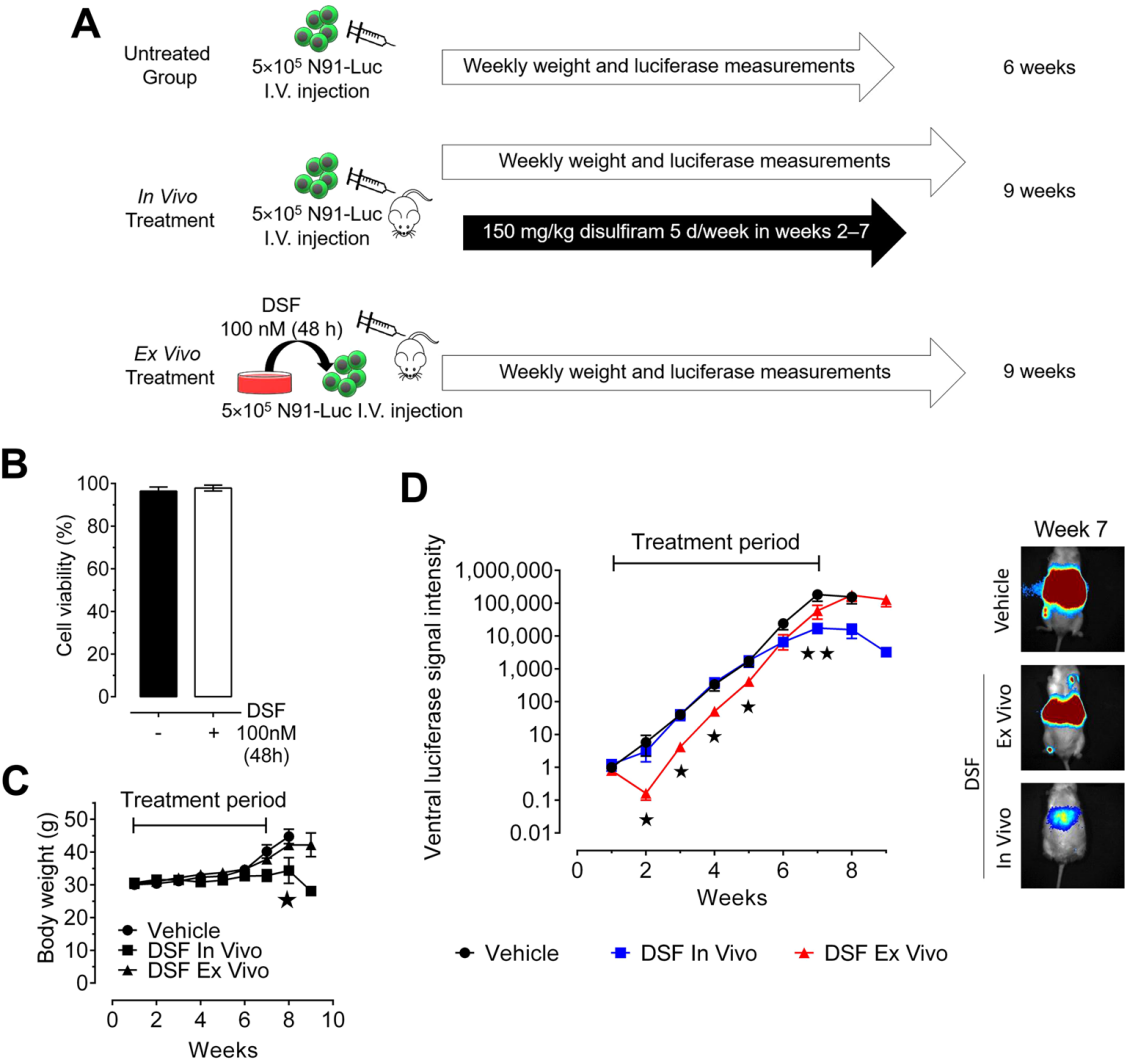
The disulfiram emulsion reduces neuroblastoma growth in vivo. (**A**) Experimental strategy for disulfiram administration in mice. Three groups of 10 NOD/SCID/IL2rγ^null^ mice were intravenously injected with 5 × 10^5^ N91-luc neuroblastoma cells into the lateral tail vein. The in vivo group received intraperitoneal injections of 150 mg/kg disulfiram 62:38 emulsion 5 days/week for 5 weeks, the vehicle group received an emulsion without disulfiram, and the ex vivo group’s N91-luc cells were pre-treated with 100 nM emulsion-based disulfiram for 48 h before injection. (**B**) The viability of N91-luc neuroblastoma cells after exposure to the vehicle and disulfiram emulsions. (**C**) The body weights of the mice over the 9 weeks of in vivo experimentation. (**D**) Left: tumor progression was measured every week by bioluminescence of N91-luc neuroblastoma cells in mice. Right: representative images of neuroblastoma cell fluorescence in the three groups during week 7. Stars indicate significant differences between the treated group and the untreated control (*p* ≤ 0.05 by unpaired Student’s *t*-test).

To evaluate the repurposing potential of disulfiram in neuroblastoma in the developed emulsion, we performed an in vivo study using immune-compromised mice. The lateral tail veins of NOD/SCID/IL2rγ^null^ mice (Fig. 6A) were intravenously injected with N91-luc neuroblasts. The vehicle and in vivo treatment groups received intraperitoneal injections of the blank and 150 mg/kg disulfiram emulsions, respectively, 5 days/week for 5 weeks. The ex vivo treatment group was injected with N91-luc cells pretreated with 100 nM disulfiram for 48 h. This group was included to assess whether disulfiram could produce long-term anticancer effects in mice and if its effects are reversible or not. Prior to injecting the pretreated N91-luc cells into the lateral tail vein, we measured their viability after treatment with blank or 100 nM disulfiram emulsions for 48 h (Fig. 6B). No differences in viability were observed between the untreated and treated cells, enabling direct comparisons between the three experimental groups.

We measured the body weights of the mice for 9 weeks during the in vivo experiment as a marker of toxicity induced by either neuroblastoma progression or the emulsions (Fig. 6C). The body weights were similar between the groups for the first 7 weeks, indicating that emulsified disulfiram does not produce significant toxicity using our dose schedule. In weeks 8 and 9, neuroblastoma tumor masses increased, resulting in increased body weights in the vehicle and ex vivo groups. However, the body weights of mice in the in vivo treatment group were significantly lower, suggesting that emulsified disulfiram delayed neuroblastoma tumor growth.

Tumor progression was measured every week by detecting the bioluminescence of the N91-luc neuroblastoma cells (Fig. 6D). Vehicle-treated mice displayed strong increases in bioluminescence, suggesting exponential growth of the N91-luc cells. Tumor growth in the in vivo group was identical to that of the vehicle group for the first 6 weeks; however, disulfiram treatment significantly reduced their bioluminescence in weeks 7–9 (i.e., 3 weeks after treatment arrest). This suggests that disulfiram treatment significantly delayed *MYCN*-amplified neuroblastoma progression. The ex vivo treatment group showed significant delays in tumor growth during the first 5 weeks, with significantly less bioluminescence than the vehicle group despite no in vivo treatment, indicating long-term effects of disulfiram initial exposure. However, for the remainder of the experiment (weeks 6–9), the bioluminescence of the ex vivo treatment group was similar to that of the vehicle group, suggesting that continuous treatment may be required to control neuroblastoma treatment in this model. Although disulfiram emulsion treatment delayed neuroblastoma progression, there was no significant difference in survival between the groups (Suppl. Fig. S5B). However, our preclinical evaluation of disulfiram emulsion demonstrated its safety and revealed an encouragingly significant delay in neuroblastoma progression.

## Discussion

Disulfiram is an immensely successful treatment for chronic alcoholism that has been prescribed for over 70 years. Prolonged treatment has negligible adverse effects, proving disulfiram’s safety. Crucially, even its intended effects on ALDH only impact the patient after alcohol consumption, making disulfiram ideal for pediatric repurposing. In addition to ALDH^29,30^, disulfiram has several other reported targets, some of which suggest repurposing potential for cancer treatment. Intriguingly, disulfiram administration improved overall survival in clinical trials of patients with lung and breast cancer^31–35^. Here, we demonstrated that low nanomolar concentrations of disulfiram had anticancer effects in multiple neuroblastoma cell lines and delayed tumor growth in vivo. Mechanistically, disulfiram dose-dependently decreased KAT2A, significantly reducing the acetylation levels of its target lysine residues on histone H3. We also observed decreased MYCN levels at low doses, possibly due to decreased KAT2A activity^19^. Interestingly, disulfiram, at low doses resulted in the onset of expression of neuronal differentiation pathways at the same low doses than MYCN and histone acetylation reduction. Neuronal differentiation may be responsible for the long-term effects observed in clonogenic assays and delayed growth observed in mice (in the ex vivo group). Higher doses may induce more immediate cytotoxicity responses, also shown in other studies^31,32,46^. This novel target of disulfiram should be confirmed in other neuroblastoma models in vitro and in vivo. Interestingly, pharmacological KAT inhibition also decreases MYCN in neuroblasts^47^, triggering the caspase-independent death of neuroblastoma cells and xenografts^22^. Unfortunately, currently available KAT inhibitors have limited potential for clinical application because of issues with selectivity, toxicity, and potency^19^. Thus, repurposing disulfiram is a promising new approach to treating pediatric neuroblastoma.

Disulfiram’s metabolic pathway and toxicological profile are well-established, which could enable rapid repurposing via clinical trials of pediatric patients with high-risk neuroblastoma^27,28^. However, disulfiram is unstable in the blood and gastric fluid and is currently administered orally as a pill, preventing its direct clinical adaptation in young children with neuroblastoma. As disulfiram is highly lipophilic, we developed a W/O emulsion, which had anticancer effects in vitro and in vivo. In addition, the disulfiram-loaded emulsion was well-tolerated by the mice, producing no significant toxicities. Altogether, our studies provide a solid rationale for repurposing disulfiram to treat high-risk neuroblastoma. Disulfiram could also synergize with chemotherapies, radiotherapies, and immunotherapies, make it an interesting drug to incorporate into next-generation clinical trials to improve the initial response rates of patients with high-risk neuroblastoma^7^. Supporting this idea, a preclinical study demonstrated that disulfiram enhanced the antitumor efficacy of external-beam γ-irradiation and ^131^I-metaiodobenzylguanidine^46^.

The need to develop safe therapies for high-risk neuroblastoma is high. Even with extensive multimodal therapies, only half of the children diagnosed with high-risk neuroblastoma survive, and half of the survivors suffer long-term adverse effects, such as hearing loss, secondary cancers, endocrinopathies, psychological and learning difficulties, and reproductive issues^7^. Repurposing safe and already approved drugs like disulfiram could fill this crucial medical need and improve and prolong the lives of children with high-risk neuroblastoma.

## Methods

### Cell culture and disulfiram treatment

The neuroblastoma cell lines IGR-N91, IMR-32, SK-N-DZ, SK-N-SH, and SK-N-AS were obtained from ATCC (Manassas, VA, USA). IGR-N91, SK-N-DZ and SK-N-AS cells were grown in Dulbecco’s modified Eagle’s medium. SK-N-SH and IMR-32 cells were grown in Eagle's minimum essential medium. Patient-derived neuroblastoma (SJ-N-TQ-24) cells, obtained from a 4-year-old boy with stage 4 neuroblastoma featuring *MYCN* amplification (50 copies) and several chromosomal aberrations (loss of 1p, 3q, and 19p; gain of 2p, 11q, and 17q without *ALK* receptor tyrosine kinase amplification); a gift from Dr. Elie Haddad (Université de Montréal) were cultured in F12K cell culture medium. All cell culture media were supplemented with 10% fetal bovine serum (Wisent, Saint-Jean-Baptiste, QC, Canada). Cells were maintained in log phase growth and passaged twice weekly. Luciferase-tagged IGR-N91 (N91-luc) cells were generated for in vivo experiments. Briefly, lentiviruses were generated by transducing 293 T cells with plasmids expressing luciferase (pHRSIN UCOE SFFV fLuc), packaging components (pMDlg/pRRE and pRSV-Rev, Invitrogen) in an envelope plasmid (pMD2.g) according to the manufacturer’s directions. Cell culture supernatants were harvested and titrated with the HIV-1 p24 ELISA Kit (Xpressbio, Frederick, MD, USA) according to the manufacturer’s instructions. IGR-N91 cells (1 × 10^5^ per well) were seeded in 6-well plates 24 h before infection, then incubated with polybrene and 100 ng viral particles overnight. After removing the polybrene-viral particle mix, the cells were incubated for 48 h, then cells with stable luciferase expression were selected with 10 µg/mL blasticidin for 2 weeks. Disulfiram (tetraethylthiuram disulfide; > 97% purity) was purchased from Acros Organics. For in vitro studies, it was dissolved in 100% dimethyl sulfoxide (DMSO) at a stock concentration of 10 mM and stored at −80 °C for up to 3 months. Its main metabolite, DDTC, was purchased from Fisher Chemical.

### Growth and viability analyses

Log-phase cells were seeded and treated with varying concentrations of disulfiram for 48 h to generate dose–response curves. The medium and drug were replaced daily during the treatment period. Viability was evaluated using Guava ViaCount Reagent (Luminex, Austin, TX, USA, 4000-0040) on a Guava EasyCyte 6HT Flow Cytometer (EMD-Millipore, Burlington, MA, USA). Half-maximal inhibitory concentration (IC_50_) values were calculated in GraphPad Prism 9 (GraphPad Software, Boston, MA, USA), using the formula log(inhibitor) response-variable slope (four parameters) and the least square method.

### Cell cycle analysis

Following treatment with DMSO or disulfiram (25, 50, or 100 nM) for 48 h, IGR-N91 cells were fixed and stained with bromodeoxyuridine (BrdU) and/or 7-amino-actinomycin (7-AAD, BD Pharmingen BrdU Flow Kit). Flow cytometry analyses were performed on a BD FACS Canto II Flow Cytometry System (Beckton Dickinson).

### Clonogenic assays

Neuroblastoma cell lines were seeded in 6-well plates (500 cells/well). The next day, they were treated for 48 h with 50–300 nM disulfiram, then incubated in drug-free media for 2 weeks to allow colony formation. The colonies were stained and fixed with 0.5% methylene blue in 50% methanol, rinsed, dried, and counted. The clonogenic potential of each line was calculated relative to untreated controls (DMSO only).

### RNA extraction and RNA sequencing

Before extracting the RNA, we used QIAshredder (#75654, QIAGEN Sciences, Germantown, MD, USA) to homogenize the cell lysates and eliminate any debris. Total RNA was extracted using an RNeasy Mini Kit (#74104, QIAGEN Sciences) according to the manufacturer’s instructions. Then, 10 μg of each purified extract was treated with DNAse to eliminate DNA contamination, and the RNA quality and yield were verified with Bioanalyzer chips using an Agilent RNA 6000 Nano Kit according to the manufacturer’s instructions. We created mRNA libraries using 25 ng of mRNA extract and the TruSEq Stranded mRNA LT Sample Prep Kit (#RS-122-2101, Illumina, San Diego, CA, USA). RNA sequencing was performed on a HiSeq 2500 System (Illumina) in “rapid run” mode. Reads were aligned to the human genome (hg19) using STAR v2.4.2^48^. Differential gene expression analysis between untreated and treated samples was performed using DESeq2. v1.10.1^49^. The threshold for differential expression was a log_2_ fold change of + 1 for upregulated genes and − 1 for downregulated genes. Metascape was used to generate GO terms to analyze the differentially expressed genes^50^. Genes with adjusted* p* values < 0.05 were included in pathway and enrichment analyses.

### Western blotting

For whole-cell protein extraction, cell pellets were lysed in cold RIPA buffer (50 mM Tris–HCL pH 7.4, 5 mM EDTA, 250 mM NaCl, 50 mM NaF, 0.1 mM Na_3_VO_4_, 0.1% Triton X-100), supplemented before use with cOmplete Protease Inhibitor Cocktail (Roche) and 5 mM sodium butyrate. For histone analysis, cell pellets were acid-extracted overnight using Abcam’s histone extraction protocol in phosphate-buffered saline containing 0.5% Triton X-100, 2 mM PMSF, 0.02% NaN_3_, and 10 mM sodium butyrate and supplemented with cOmplete Protease Inhibitor Cocktail before use. The protein concentrations of whole-cell and histone extracts were measured using the Bradford assay. Proteins were resolved by sodium dodecyl sulfate–polyacrylamide gel electrophoresis and transferred to polyvinylidene difluoride membranes (Bio-Rad) for western blotting. The following antibodies were used in western blots of whole-cell extracts: actin (Sigma-Aldrich #A2228; 1:1000), KAT2A (Santa Cruz Biotechnology #sc-365321; 1:1000), KAT2B (Cell Signaling Technology #3378; 1:1000), KAT3A (Cell Signaling Technology #7839; 1:5000), KAT3B (Active Motif #61402; 1:5000), KAT5 (Abcam #ab137518; 1:2500), KAT6A (Active Motif #39868; 1:1000), KAT7 (Bethyl Labs #A302-224A-T; 1:1000), HDAC1 (Cell Signaling Technology #5356; 1:5000), histone deacetylase 2 (HDAC2; Cell Signaling Technology #5113; 1:1000), and MYCN (Millipore #MABE333; 1:1000). The following antibodies (all from Active Motif) were used in western blots of acid-extracted histones: H3 total (#39763; 1:5000), H3K9 acetyl (#39917; 1:5000), H3K14 acetyl (#39698; 1:2500), H3K27 acetyl (#39134; 1:2500), H3K27 dimethyl (#61435; 1:1000), H3K27 trimethyl (#39155; 1:5000), H3K9 dimethyl (#39753; 1:5000), and H3K9 trimethyl (#39765; 1:5000). Signals were detected using Clarity Western ECL Substrate (Bio-Rad) and digitized with an ImageQuant LAS 4000 imager (GE Healthcare). Band intensities were quantified with ImageJ (National Institutes of Health, Bethesda, MD, USA) using actin or H3 as a loading controls, and statistically significant differences were determined by one-way analysis of variance in GraphPad Prism. Full western blot images are shown in Suppl. Fig. S6.

### Emulsion analyses

Soybean oil was obtained from Alfa Aesar (Haverhill, MA, USA). Ultrapure water produced by a Milli-Q system (Sigma-Aldrich Canada, Oakville, ON, Canada) was used in all experiments. Span 80 and Tween 80 were purchased from Sigma-Aldrich Canada. Viscosity was measured using a Brookfield DV-III Ultra Programmable Rheometer (Brookfield Engineering, Middleboro, MA, USA) with a CP51 spindle and a 25 mL sample volume. Physical stability includes an invariance of rheological behavior. The rheological properties of an emulsion constitute one of the best means of studying the influence of formulation parameters and manufacturing processes, but also a method of controlling the reproducibility of production and conservation^44^. Particles were imaged on an Zeiss ApoTome 2.0 microscope with Axio ZoomV.16 and an AxioCam Camera (Zeiss, Oberkochen, Germany) and measured with Zen 2.5 Pro software (Zeiss). Stability and release studies were performed by HPLC using a Shimadzu instrument coupled to a UV detector (Shimadzu Scientific Instruments, Columbia, MD, USA) and a Hypersil PFP 5 μm C18 150 Å 50 × 4.60 mm analytical column (Thermo Scientific, San Jose, CA, USA).

### Disulfiram emulsion formulation

Disulfiram (maximal concentration: 38 mg/mL) was dissolved in soybean oil by gentle heating (at 32 °C for 2 min), vortexed for 1 min, and filter-sterilized. Emulsifiers Tween 80 (Fisher) and Span 80 (Sigma-Aldrich) were filter-sterilized and mixed by magnetic agitation for 20 min before adding disulfiram in oil and agitating for another 10 min. Sterile Milli-Q water was added to the disulfiram/oil/surfactant mixture and vortexed vigorously for 1 min 50 s to yield a W/O disulfiram emulsion (with a ratio of 1:0.02:0.15:0.62 for disulfiram in oil, Tween 80, Span 80, and water). A blank emulsion containing soybean oil without disulfiram was used as a vehicle control.

### Chemical stability

HPLC–UV was used to study the chemical stability of disulfiram, to detect degradation products and monitor in vitro release. Separation was performed with a Hypersil PFP 5 μm C18 150 Å 50 × 4.60 mm analytical column with isocratic elution in a mobile phase of 30% acetate buffer (0.05 M KH_2_PO_4_, pH 7.0) and 70% methanol (flow rate: 1 mL/min; detection wavelength: 250 nm). A calibration curve was prepared with disulfiram standards (5–20 µg/mL). All initial dilutions were prepared in DMSO, then the injection samples were diluted in the mobile phase (70% methanol). Samples (20 µL) were prepared and injected in triplicate, and their results were compared to the concentrations of the corresponding disulfiram standards. Chemical stability analyses were performed over 30 days at 4 °C and at RT.

### In vitro release studies

Disulfiram release was studied from the W/O emulsion to an aqueous phase called receptor fluid. Then, gently stirred with a magnetic bar rotating at 100 rpm. The measure was based on membrane technology and transport of disulfiram through a dialysis membrane by solution-diffusion mechanism, diffusing from interior part of the membrane to its surface. 100 mL of phosphate buffer pH 7.4 containing 10 mg/mL of bovine serum albumin (BSA) was used as receptor fluid and a Spectra/Por Dialysis Membrane Tubing 1500 Dalton MWCO as dialysis membrane. 1 g of emulsion was placed inside the membrane. The full glassware device was at room temperature. At each timepoint (15 min, 30 min, 1 h, 2 h, 3 h, 4 h, 6 h, 8 h, 16 h, 18 h, 20 h, 22 h, 24 h), 1 mL of receptor fluid was collected and replaced by 1 mL of fresh medium. Disulfiram concentrations were determined by HPLC–UV as above.

### Animal experiments

NOD/SCID/IL2rγ^null^ mice acquired from The Jackson Laboratory (Bon Harbor, ME, USA) were bred and maintained under pathogen-free conditions. At 7–9 weeks old, 5 × 10^5^ N91-luc neuroblasts were injected into the lateral tail veins of 30 male mice, which were divided into three groups of 10. The in vivo group received intraperitoneal injections of 150 mg/kg disulfiram emulsion 5 days per week, the vehicle group received the emulsion without disulfiram, and the ex vivo group was injected with N91-luc cells that had been pretreated with 100 nM disulfiram for 48 h. Tumor growth and body weight were measured weekly. Tumor progression was measured by bioluminescence imaging on a LabeoTech trans fluorescence imager after intraperitoneal injection of luciferine (3.6 mg in 120 µL, Perkin-Elmer), once a week until mice reached a clinical endpoint (reduced mobility and/or abdominal distension). Mice were anesthetized by isoflurane inhalation (2% in 2 L/min oxygen) before imaging and monitored for complete recovery after each procedure. Fluorescence signals were quantified using ImageJ and analyzed using GraphPad Prism. Mice were maintained at the animal facility of CHU Sainte-Justine Research Center in accordance with the protocol approved by the Institutional Animal Care and Use Committee (*Comité institutionnel des bonnes pratiques animales en recherche*, CIBPAR#527). All methods were performed in accordance with relevant guidelines and regulations and are reported in accordance with the Animal Research: Reporting of In Vivo Experiments guidelines (https://arriveguidelines.org). After sacrifice, intraperitoneal fluid samples (~ 20 µL) were centrifuged to separate the oil phase, which was analyzed by HPLC–UV to quantify the remaining disulfiram. Metabolites in the samples were identified by HR-MS^45^.

### Supplementary Information

Supplementary Information.

## Data Availability

Transcriptomic data generated during this study are publicly available in the GEO database under accession number GSE226162.
