# Supplementary material for: Repurposing disulfiram, an alcohol-abuse drug, in neuroblastoma causes KAT2A downregulation and in vivo activity with a water/oil emulsion
Source: Sci Rep. 2023 Sep 30;13:16443. doi: 10.1038/s41598-023-43219-2 (PMC10543387; doi:10.1038/s41598-023-43219-2)
Supplement: Supplementary file 1 — Supplementary Information. [file 41598_2023_43219_MOESM1_ESM.pdf]

SUPPLEMENTARY FIGURE LEGENDS

Supplementary figure S1: Disulfiram effects on neuroblastoma cell lines

(A) Cell cycle analysis using BrdU staining analyzed by flow cytometry in SK-N-SH cells treated with disulfiram for 48 h at concentrations of 25, 50 and 100 nM (*n* = 2). (B) Representative images of neurite outgrowth in N91 colonies, two weeks after disulfiram treatment (50 nM for 48 h) as compared to colonies of untreated N91 cells. A graphical representation shows the percentage of N91 cells with and without neurites from untreated and treated colonies (100 cells were counted per group). (C) GSEA enrichment plot of Hallmark G2/M checkpoint genes in untreated and disulfiram treated (50 nM for 48 h) N91 cells. (D) Top 10 downregulated GO terms in N91 cells after disulfiram treatment (50 nM for 48 h) in N91 cells and their normalized enrichment scores ($\log_2$ fold change < -1; adjusted *p* value < 0.05).

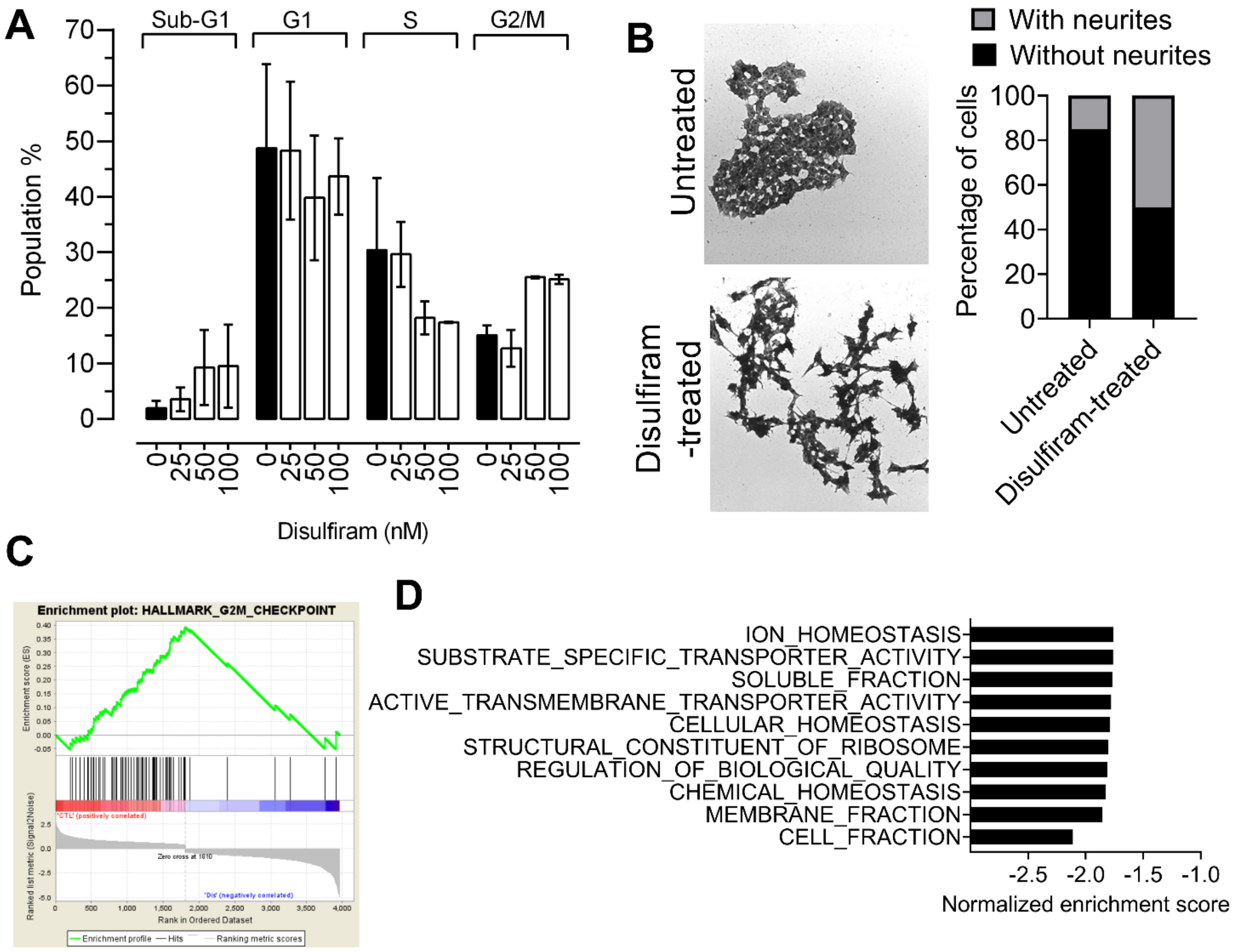

Supplementary figure S2: Disulfiram effects on HDACs, histone methylation and MYCN levels.

(A-B) Disulfiram effects on histone deacetylase protein levels and histone post-translational modifications. *MYCN*-amplified N91 neuroblastoma cells were exposed to disulfiram treatment at 50, 100, and 200 nM for 48 h. (A) Protein expression levels of histone deacetylase 1 (HDAC1) and 2 (HDAC2). Percentage of expression is normalized to actin levels and expressed relative to untreated cells (n = 3). (B) Post-translational modifications in histone H3 methylation levels on lysine residues 9, and 27 and their quantification relative to H3 total levels and untreated cells (n = 3). (C) *MYCN* transcript (RPKM- Reads Per Kilobase Million) levels in N91 cells, untreated and treated with disulfiram (50 nM, 48 h) in RNA-sequencing data set (* indicates $p < 0.05$, paired t-test, n = 3).

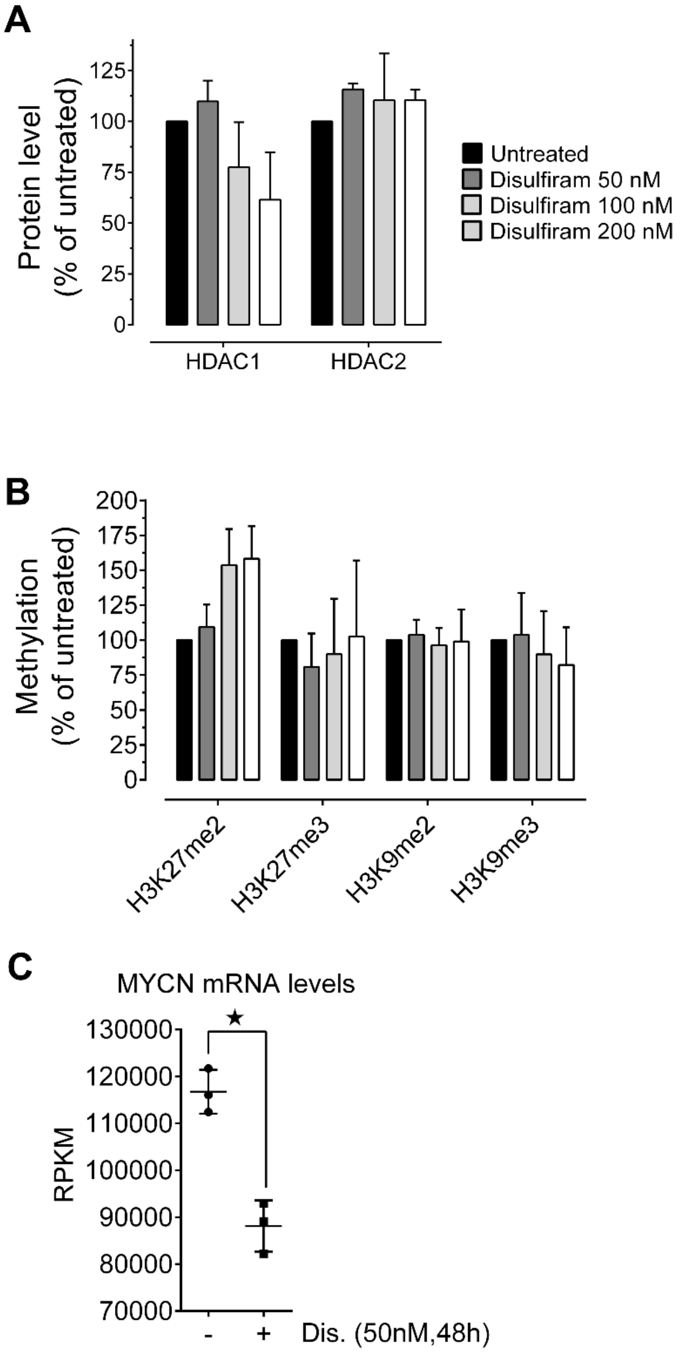

Supplementary figure S3: Optimization of emulsions.

Oil-water ratio optimization at (A) 4ºC and (B) room temperature using 4 different oil:water emulsions.

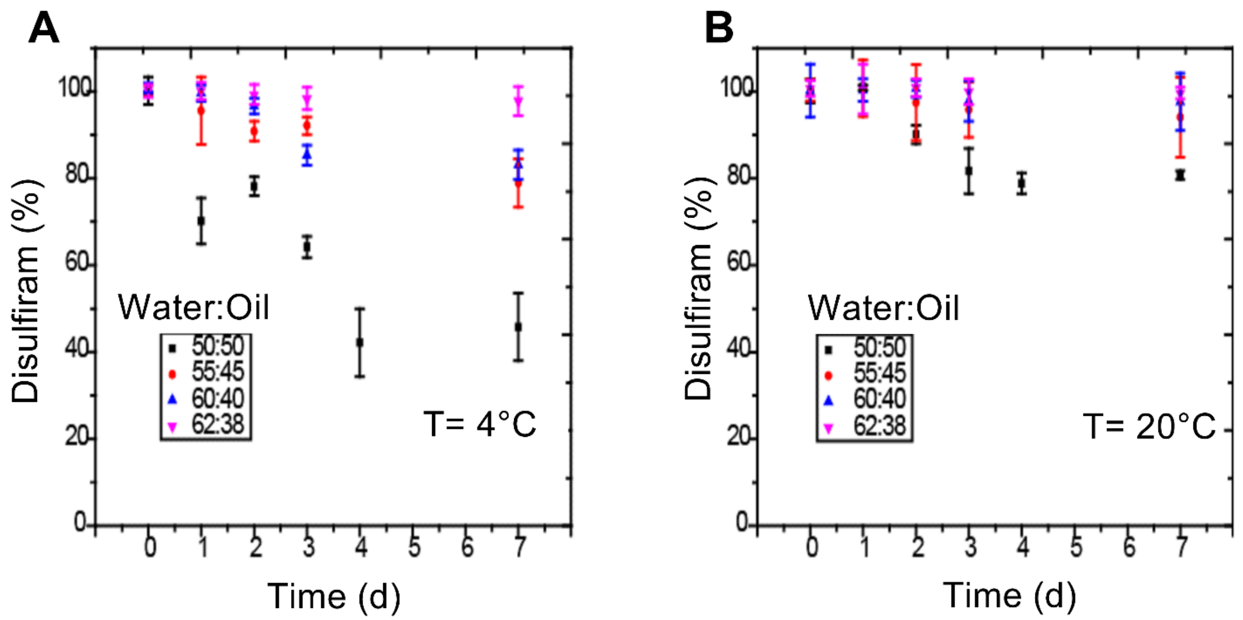

Supplementary figure S4: Stability of disulfiram loaded formulations

(A) Visual stability of 4 blank formulations and 1 disulfiram loaded formulation (38 mg/mL) during 7 d at 4ºC (n = 3). (B) Visual stability of the 62:38 emulsion without (blank) and with disulfiram during 3 d at 4ºC and 22 ºC. (C) Particle size of the 62:38 blank emulsion and 62:38 loaded emulsion with disulfiram using optical microscopy. The number of particles was assessed using ImageJ and their sizes determined by optical microscopy (n = 3).

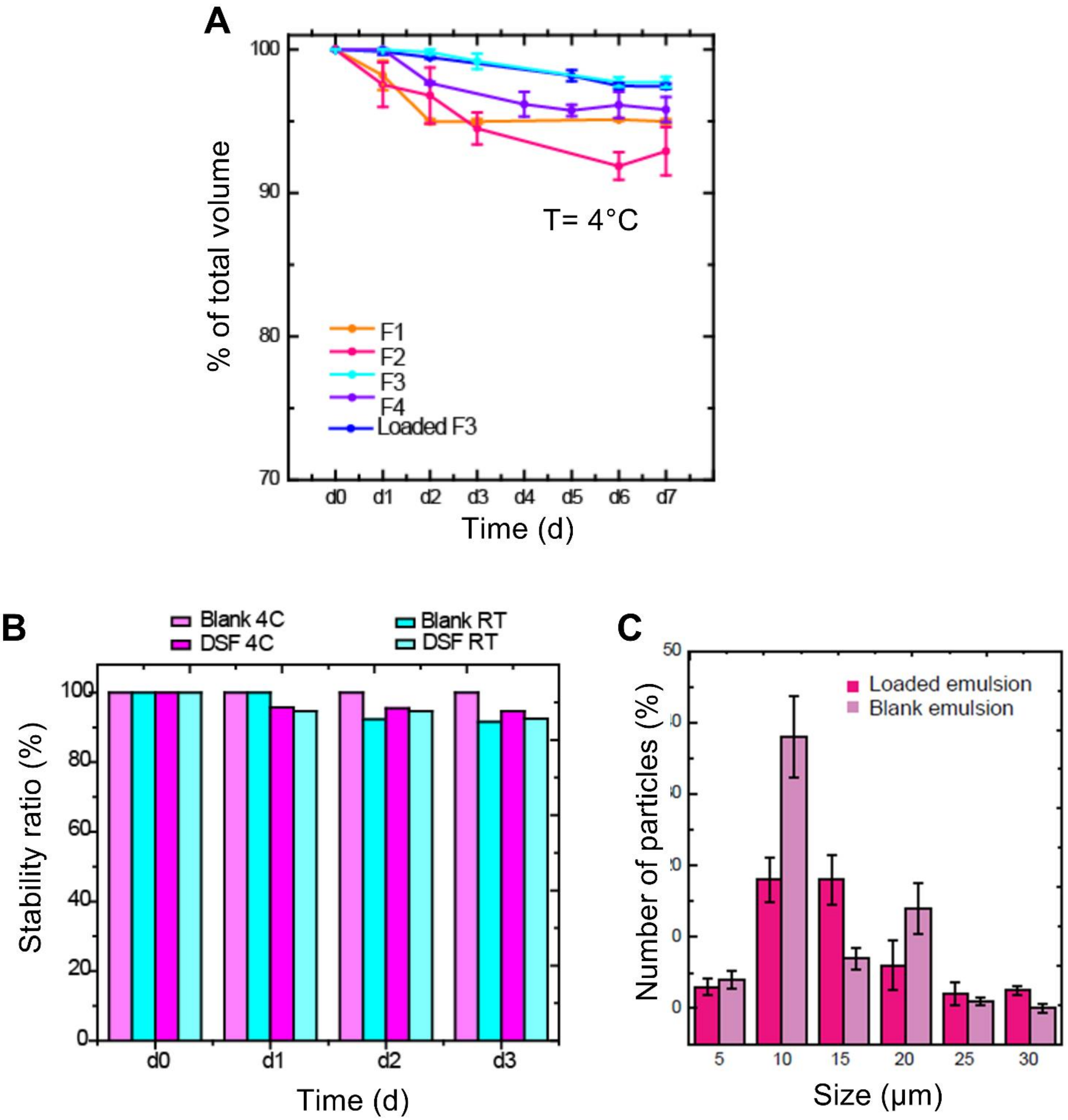

Supplementary figure S5: Measurements of the viscosity of the emulsions.

(A) Rheological curves were generated with blank formulations F1, F2, F3, F4 and with formulation F3 loaded with disulfiram at room temperature. (B) Kaplan-Meier survival curves of NOD-scid IL2Rγnull (NSG) mice transplanted with 5 x10^5 N91-luc neuroblastoma cells, intravenously into the lateral tail vein. Three experimental groups are shown: 1) untreated controls (vehicle only), 2) Disulfiram *in vivo* treatment (intraperitoneal injections of 150 mg/kg disulfiram in 62:38 emulsion, 5 days per week for 5 weeks), and 3) *Ex-Vivo* group where N91-Luc cells were pre-treated *Ex-Vivo* for 48 h with 100 nM disulfiram (in the emulsion) prior to injection in mice.

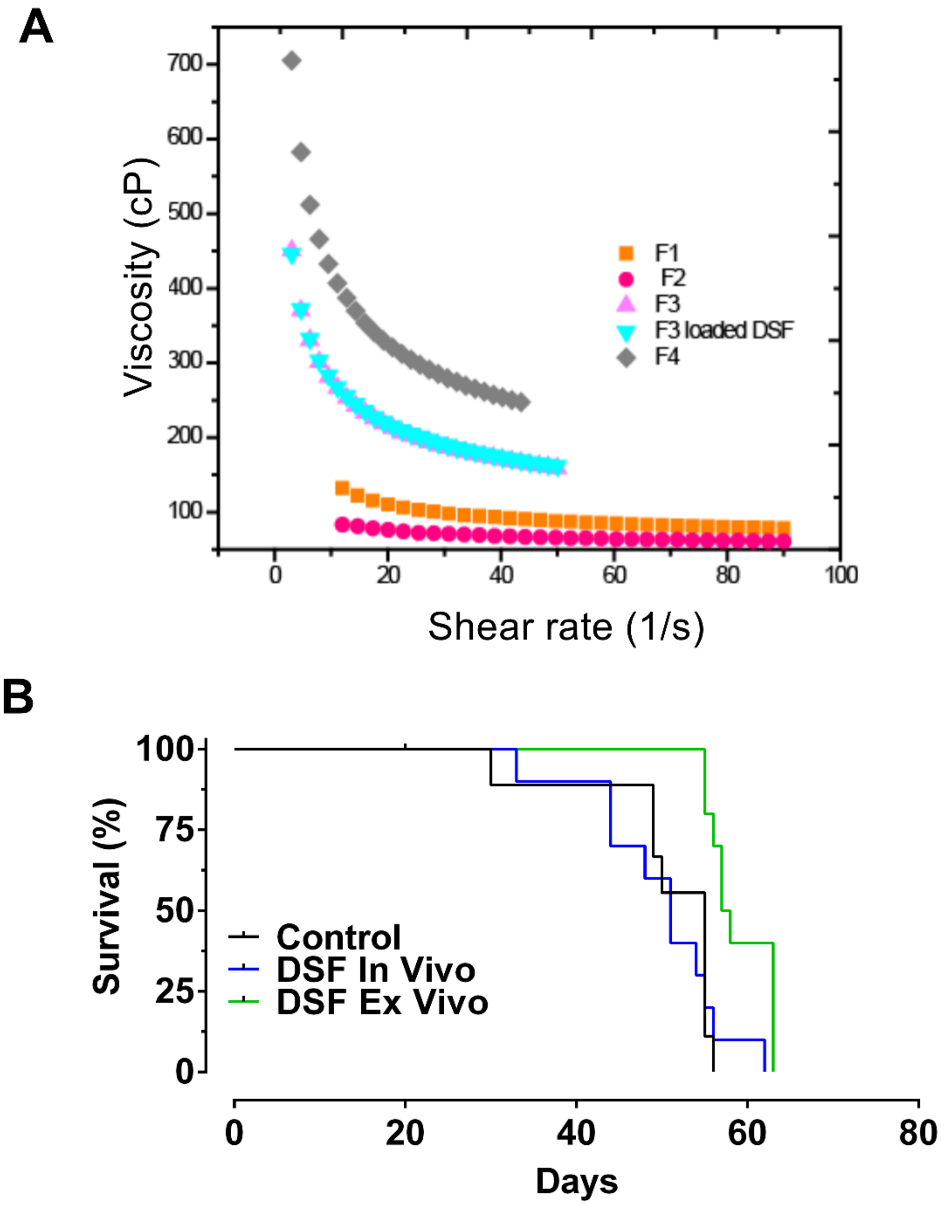

Supplementary figure S6: Full western blot images

(A) Representative blot images of KAT2A (GCN5) and actin presented in Figure 3A. (B) Blot images of H3K27Ac, H3K14Ac, H3K9Ac and H3 presented in Figure 3C. (C) Blot images of MYCN and actin presented in Figure 3D. (D) Blot images of MYCN and actin presented in Figure 5B. (E) Blot images of H3H9me2, H3K9me3, H3K27me2, H3K27me3 and total H3 whose quantification is shown in supplementary Fig.S2B.

A)

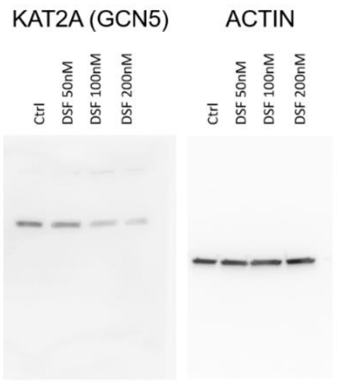

B)

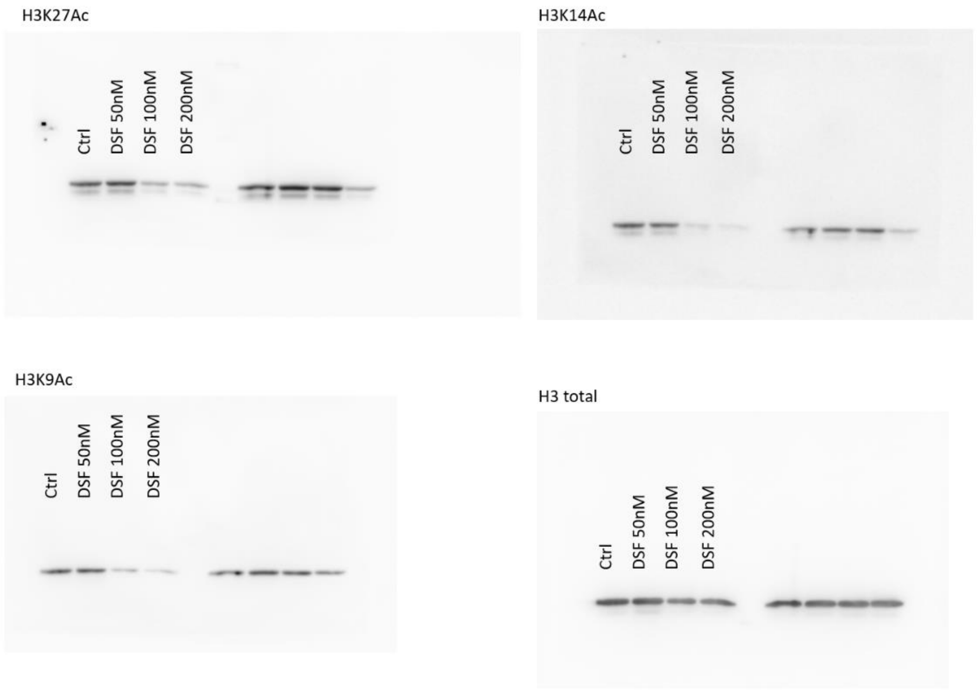

C)

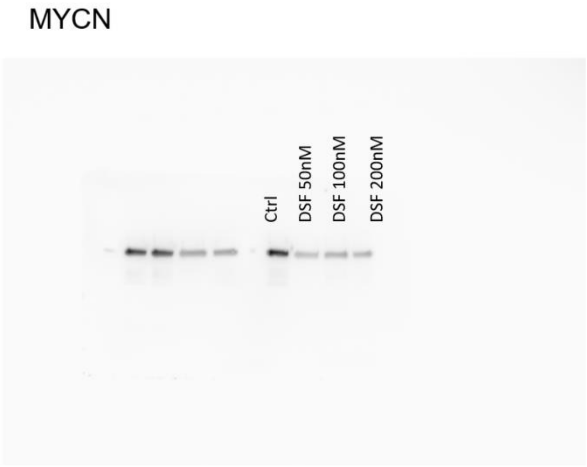
MYCN
Ctrl
DSF 50nM
DSF 100nM
DSF 200nM

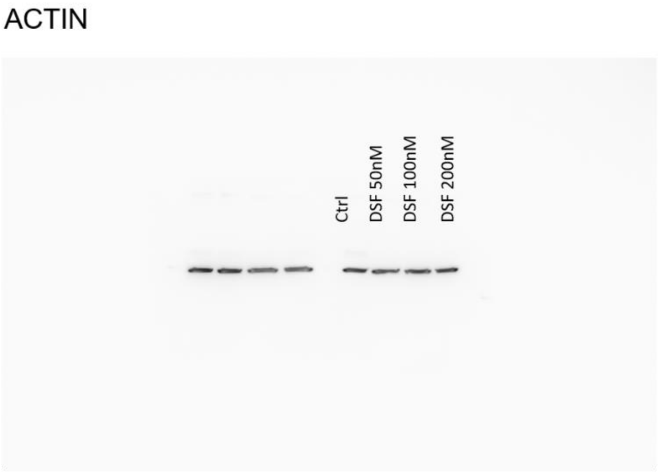
ACTIN
Ctrl
DSF 50nM
DSF 100nM
DSF 200nM

D)

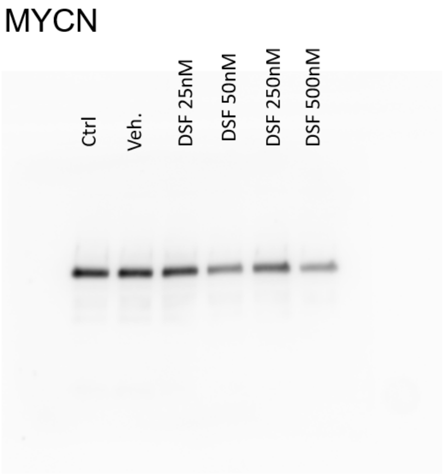
MYCN
Ctrl
Veh.
DSF 25nM
DSF 50nM
DSF 250nM
DSF 500nM

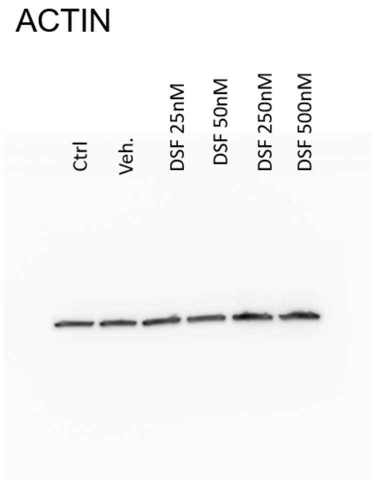
ACTIN
Ctrl
Veh.
DSF 25nM
DSF 50nM
DSF 250nM
DSF 500nM

E)

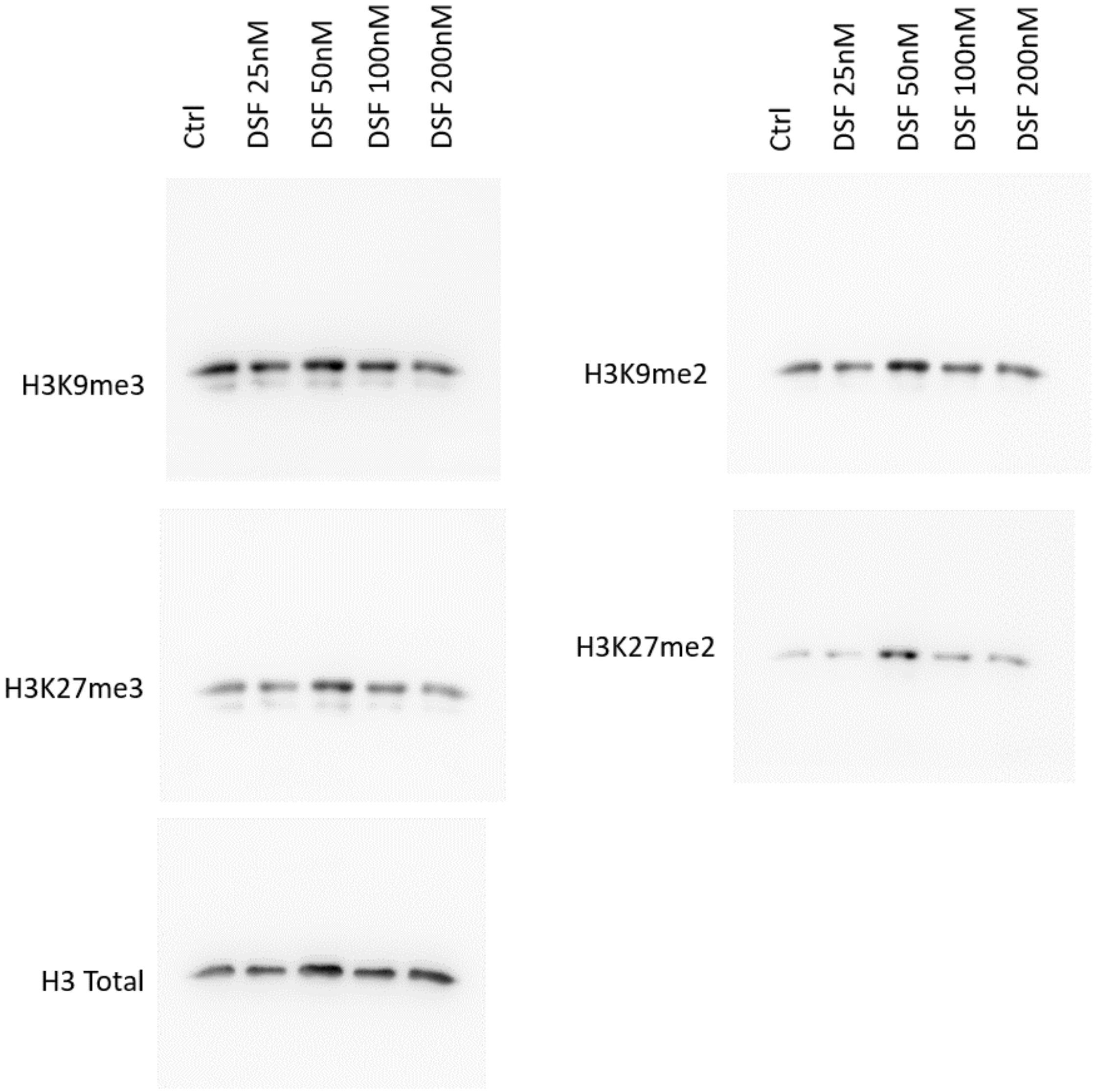
Ctrl
DSF 25nM
DSF 50nM
DSF 100nM
DSF 200nM
H3K9me3
H3K27me3
H3 Total
Ctrl
DSF 25nM
DSF 50nM
DSF 100nM
DSF 200nM
H3K9me2
H3K27me2

Supplementary table S1: MYCN target genes were curated in the ChIP Enrichment Analysis database. RNA-seq data were compared using the Harmonizome website. Six genes were significantly downregulated (log2 fold change < -1, adjusted *p* value < 0.05) and 52 more moderately downregulated (log2 fold changes between -0.5 and -1, adjusted *p* value < 0.05) after disulfiram treatment (50 nM; 48 h) in N91 cells.

Gene Symbol	Description	Log2FC	Adjusted *p* value
CFAP45	cilia and flagella associated protein 45	-1,624	0,03220965
RERG	RAS like estrogen regulated growth inhibitor	-1,529	2,31E-26
SSTR2	somatostatin receptor 2	-1,044	9,68E-18
SOCS3	suppressor of cytokine signaling 3	-1,009	2,11E-11
FABP5	fatty acid binding protein 5	-1,002	0,04621618
PIK3R2	phosphoinositide-3-kinase regulatory subunit 2	-1	0,0412963
HNRNPDL	heterogeneous nuclear ribonucleoprotein D like	-0,792	1,21E-12
CPXM1	carboxypeptidase X, M14 family member 1	-0,752	1,10E-06
SLC2A6	solute carrier family 2 member 6	-0,75	3,32E-07
SRSF1	serine and arginine rich splicing factor 1	-0,731	5,03E-10
TERT	telomerase reverse transcriptase	-0,724	0,01421833
TOP1	DNA topoisomerase I	-0,711	0,00063785
LFNG	LFNG O-fucosylpeptide 3-beta-N-acetylglucosaminyltransferase	-0,709	0,01230699
NASP	nuclear autoantigenic sperm protein	-0,706	3,41E-05
RECQL4	RecQ like helicase 4	-0,706	2,14E-05
CDH18	cadherin 18	-0,705	1,39E-08
ACAP3	ArfGAP with coiled-coil, ankyrin repeat and PH domains 3	-0,699	2,84E-08
AMDHD2	amidohydrolase domain containing 2	-0,69	6,61E-05
MC4R	melanocortin 4 receptor	-0,688	0,02489987
SEMA3G	semaphorin 3G	-0,679	0,0009865
CYB561	cytochrome b561	-0,67	1,57E-13
B4GALNT4	beta-1,4-N-acetyl-galactosaminyltransferase 4	-0,667	0,00020888
MXD4	MAX dimerization protein 4	-0,658	0,00040436
C1orf159	chromosome 1 open reading frame 159	-0,656	0,00064563
HMGB3	high mobility group box 3	-0,652	2,11E-11
HNRNPH3	heterogeneous nuclear ribonucleoprotein H3	-0,644	0,00120653
ALDH7A1	aldehyde dehydrogenase 7 family member A1	-0,643	0,00142863
WDR34	WD repeat domain 34	-0,639	2,62E-12
JUNB	JunB proto-oncogene, AP-1 transcription factor subunit	-0,638	0,00570245
FKBP5	FKBP prolyl isomerase 5	-0,636	0,007918
HNRNPM	heterogeneous nuclear ribonucleoprotein M	-0,628	1,12E-05
COL11A2	collagen type XI alpha 2 chain	-0,615	0,03566356
SRSF10	serine and arginine rich splicing factor 10	-0,612	9,63E-10
SGSM2	small G protein signaling modulator 2	-0,605	2,91E-05
SLC16A1	solute carrier family 16 member 1	-0,601	2,68E-06
NCL	nucleolin	-0,598	0,00315716
MXD3	MAX dimerization protein 3	-0,585	0,00700318
PPP5C	protein phosphatase 5 catalytic subunit	-0,58	0,00833953
CTDP1	CTD phosphatase subunit 1	-0,572	0,0376581
SEMA6A	semaphorin 6A	-0,57	1,21E-05

PIH1D1	PIH1 domain containing 1	-0,569	1,46E-05
PKM	pyruvate kinase M1/2	-0,566	0,01799632
HSP90AA1	heat shock protein 90 alpha family class A member 1	-0,564	0,00019689
LIG1	DNA ligase 1	-0,562	0,00163699
SERBP1	SERPINE1 mRNA binding protein 1	-0,562	0,00136882
TP53INP1	tumor protein p53 inducible nuclear protein 1	-0,561	7,14E-08
DDX5	DEAD-box helicase 5	-0,547	0,00053135
SIVA1	SIVA1 apoptosis inducing factor	-0,545	0,0039209
MCM3	minichromosome maintenance complex component 3	-0,545	8,84E-06
MAP2K6	mitogen-activated protein kinase kinase 6	-0,539	2,21E-07
YBX1	Y-box binding protein 1	-0,533	0,01795501
SNN	stannin	-0,526	0,00049335
WDR4	WD repeat domain 4	-0,52	0,00719352
ABCF1	ATP binding cassette subfamily F member 1	-0,516	0,00040001
H2AFY	H2A histone family member Y	-0,512	0,00668244
THOP1	thimet oligopeptidase 1	-0,502	0,0087192
DBI	diazepam binding inhibitor, acyl-CoA binding protein	-0,502	0,00090012
RRM2	ribonucleotide reductase regulatory subunit M2	-0,501	2,21E-07
